# Technical strategies for radiation dose optimisation in paediatric pelvic radiography: a systematic review

**DOI:** 10.1007/s12194-026-01055-9

**Published:** 2026-05-15

**Authors:** Koustubh Kamath, Suresh Sukumar, Leena R. David, Rajagopal Kadavigere, Hitesh Hasmukhlal Shah, Nitika C. Panakkal, Abhimanyu Pradhan, Winniecia Dkhar, Hari Prakash Planiswamy, Sneha Ravichandran, Dilip Shettigar, Sathya Sabina Muthu

**Affiliations:** 1 Department of Medical Imaging Technology, Manipal College of Health Professions, Manipal Academy of Higher Education, Manipal, India; 2 Department of Medical Diagnostic Imaging, College of Health Sciences, University of Sharjah, Sharjah, United Arab Emirates; 3 Radio-diagnosis & Imaging, Department of Radio Diagnosis & Medical Imaging, Kasturba Medical College, Manipal Academy of Higher Education, Manipal, Karnataka India; 4 Department of Paediatric Orthopaedics, Kasturba Medical College, Manipal Academy of Higher Education, Manipal, Karnataka India; 5 Department of Speech and Hearing, Manipal College of Health Professions, Manipal Academy of Higher Education, Manipal, Karnataka India

**Keywords:** Paediatric pelvic radiography, Radiation dose optimisation, Image quality, Automatic exposure control, High kVp technique, Source-to-image distance, Filtration

## Abstract

Paediatric pelvic radiography is a frequently performed diagnostic procedure that exposes radiosensitive organs to ionising radiation. Optimisation of imaging parameters is essential to minimise radiation dose while preserving diagnostic image quality. This systematic review aimed to evaluate technical strategies for radiation dose optimisation in paediatric pelvic radiography and assess their impact on radiation dose and image quality. A systematic search of PubMed, Scopus, Embase, and Web of Science was conducted for studies published between 2004 and 2025. Experimental and clinical studies assessing radiation dose and image quality in paediatric pelvic radiography were included. Data on exposure parameters, dose metrics, and image quality assessment methods were extracted and synthesised narratively. Nine studies met the inclusion criteria, predominantly comprising phantom-based experimental designs. Increasing tube potential (kVp), extending source-to-image distance (SID), and applying additional aluminium or copper filtration consistently reduced radiation dose. Dose reductions of up to 60–70% were reported without clinically significant loss of image quality. Although objective image quality metrics (e.g., CNR, SNR) occasionally declined, subjective assessments confirmed that images remained diagnostically acceptable. Optimisation of technical parameters in paediatric pelvic radiography can achieve substantial radiation dose reduction while maintaining clinically acceptable image quality. However, the predominance of phantom-based evidence highlights the need for further patient-based clinical studies to support routine implementation.

## Introduction

### Background

X-rays are a form of ionising radiation that have become indispensable in modern healthcare, serving as the cornerstone of diagnostic imaging in hospitals and clinics worldwide [1–3]. Their widespread adoption stems from the ability of radiographic techniques to generate high-resolution images that provide clinicians with essential diagnostic information for accurate disease detection and management across a wide spectrum of medical conditions [4].

Conventional pelvic radiography (pelvic X-ray) is one of the most performed examinations for visualising the bones and joints of the pelvis. It is routinely employed as the first-line investigation in the evaluation of suspected fractures, dislocations, or other abnormalities in this anatomical region [5]. Pelvic radiography remains the primary modality for assessing the osseous structures of the pelvic girdle and hips in both adults and children, where the anatomical complexity of overlapping bone structures complicates interpretation. To assist radiologists in systematic analysis, interpretation strategies such as the “lines, arcs, and stripes” method have been developed [6]. Although specialised pelvic projections are generally limited to problem-solving scenarios and requested by orthopaedic specialists, achieving optimal accuracy and image quality in routine projections remains vital for effective clinical management.

The clinical importance of pelvic radiography is especially evident in paediatric populations due to higher rates of trauma, developmental disorders, and anatomical differences compared to adults. Children are more susceptible to pelvic injuries from falls, sports trauma, and road traffic accidents, making radiography an essential component of trauma assessment [7]. In addition, conditions such as developmental dysplasia of the hip (DDH), Perthes disease, and slipped capital femoral epiphysis require radiographic evaluation for diagnosis, treatment planning, and follow-up [8]. The unique composition of the paediatric pelvis, comprising both ossified and cartilaginous structures, presents diagnostic challenges that demand precise imaging to avoid misinterpretation [9]. These challenges are compounded by the need for specialised immobilisation techniques to minimise motion artefacts, which are especially problematic in children, and to ensure diagnostic image quality without repeat exposures [10]. Early and accurate identification of fractures and developmental abnormalities is critical, as timely treatment significantly reduces the risk of long-term mobility impairment, deformity, or chronic pain.

Despite these benefits, the clinical utility of radiography must be weighed against the risks associated with ionising radiation. Children are known to demonstrate heightened radiosensitivity, with a longer lifetime risk horizon in which stochastic effects may manifest [11, 12]. This increased susceptibility arises from their rapidly dividing tissues and extended post-exposure lifespan, allowing greater potential for radiation-induced malignancies to develop. Epidemiological evidence from paediatric medical imaging, particularly computed tomography (CT), has demonstrated that even low-dose diagnostic exposures can be associated with a small but measurable increase in cancer risk [13, 14]. Although the absolute risk from individual radiographic examinations is low, repeated exposures may contribute cumulatively to lifetime cancer risk in children [15]. This understanding underpins the fundamental radiation protection principle of ALARA (As Low As Reasonably Achievable), which emphasises minimising dose while maintaining adequate diagnostic image quality.

In paediatric imaging, adherence to the ALARA principle requires careful protocol optimisation to balance diagnostic needs against radiation risks [16, 17]. Optimisation strategies begin with an appropriate justification of examinations to prevent unnecessary exposures [18, 19]. When justified, optimisation focuses on radiation dose reduction while ensuring diagnostic image quality, particularly avoiding repeat exposures through effective immobilisation techniques [20–22]. Critical to this process is the selection of optimised technical parameters, such as tube potential (kVp), tube current–time product (mAs), source-to-image distance (SID), filtration, AEC chamber selection, and screen/detector systems, all of which directly affect both dose and image quality.

Therefore, the objective of this systematic review is to evaluate and synthesise the available evidence on dose optimisation strategies in paediatric pelvic radiography, with a specific focus on balancing radiation safety and diagnostic integrity. Unlike broader reviews that consider general radiographic dose optimisation or multiple anatomical sites, this review uniquely concentrates on the paediatric pelvis, providing insights tailored to this vulnerable patient group and highlighting evidence-based approaches to enhance patient protection while maintaining diagnostic excellence.

## Methods

### Protocol registration and reporting

This systematic review was conducted and reported in accordance with the PRISMA 2020 guidelines [23]. The review protocol was also registered with PROSPERO.

### Eligibility criteria

In this systematic review, we concentrated on studies examining dose optimisation strategies in pelvic radiography, with particular emphasis on the paediatric population. Our inclusion criteria encompassed experimental studies and observational studies, including phantom-based experimental studies, prospective cohort studies, and cross-sectional studies evaluating radiation dose and image quality in paediatric pelvic radiography. However, most of the studies identified were experimental in nature and predominantly conducted using phantoms, with a limited number involving human participants. Studies that were purely technical reports, case reports, or those not addressing paediatric pelvic radiography and dose optimisation were excluded. The eligible studies investigated optimisation techniques such as variations in tube potential (kVp), tube current-time product (mAs), source-to-image distance (SID), the use of automatic exposure control (AEC), and the application of additional filtration. To qualify for inclusion, studies were required to report at least one quantitative measure of radiation dose, such as entrance surface dose (ESD) or effective dose (ED), and to assess image quality using either objective metrics (e.g., signal-to-noise ratio (SNR), contrast-to-noise ratio (CNR) or subjective methods (e.g., visual grading analysis).

### Information sources

Four major electronic databases, PubMed, Scopus, Embase, and Web of Science, were systematically searched for relevant studies published between 2004 and 2025. All retrieved records were screened following a structured and systematic approach. The remaining potentially eligible articles underwent further screening, including full-text review, to determine final inclusion in the systematic review.

### Search strategy

A comprehensive search strategy was developed to identify relevant literature about paediatric pelvis radiography and dose optimisation techniques. The strategy involved combining key concept groups using Boolean operators (AND/OR). The primary search terms included variations of the anatomical and imaging focus: (“pelvis radiography” OR “pelvis x-ray”) AND population terms: (“pediatric” OR “paediatric”). To capture dose optimisation techniques, terms such as (“10 kVp rule” OR “high kVp technique” OR “15percentage % rule” OR “standard technique”) were included. Imaging modality and dose-related terms were also incorporated: (“radiography” OR “digital radiography”) AND (“AEC” OR “automatic exposure control”) AND (“radiation dose” OR “effective dose” OR “image quality”). These combinations ensured a comprehensive capture of studies addressing technique modification, image quality, and radiation dose in paediatric pelvis radiography. To verify the completeness of the search strategy, the final keyword combinations were cross-checked against previous systematic reviews in related radiography domains, and citation tracking (backward and forward searching) was performed to ensure that no relevant studies were missed.

### Selection process

Two independent reviewers conducted a systematic screening of all retrieved records using a two-stage process supported by Rayyan, a web-based platform for systematic reviews(https://new.rayyan.ai/). In the first stage, titles and abstracts were screened based on predefined inclusion and exclusion criteria. Studies deemed potentially eligible proceeded to the second stage, which involved a full-text review. Any disagreements between the reviewers at either stage were resolved through discussion, and when necessary, a third reviewer was consulted to reach a consensus. The study selection process was documented using a PRISMA flow diagram, with reasons for full-text exclusions recorded and categorised.

### Data collection process

Data from the included studies were independently extracted by two reviewers using a standardised data extraction form developed in Microsoft Excel. Any discrepancies in the extracted data were resolved through discussion between the reviewers, and if consensus could not be achieved, a third reviewer was consulted. In cases where data were unclear or missing, attempts were made to contact the corresponding authors via email for clarification.

### Data items

We extracted key information from each included study, including the first author, year of publication, country, study design, and study objectives. Since most of the studies used phantoms, participant details were limited to phantom type and region of interest. Technical data, such as the number of systems, kVp, mAs, use of AEC, and dose optimisation techniques, were collected. Outcome measures included image quality and radiation dose metrics like ESD, ED, and DAP. Where available, details of statistical analysis were also noted.

### Study risk of bias assessment

Two independent reviewers assessed the risk of bias for each included study using a methodology specifically tailored to phantom-based experimental radiographic research. As the majority of included studies were phantom-based experimental investigations, with only limited human participant studies, conventional risk-of-bias assessment tools such as RoB 2.0 or ROBINS-E were not universally applicable. Instead, the risk of bias was evaluated using an adapted version of the QUADAS-2 framework. This approach focused on key areas relevant to experimental imaging studies, including the selection and setup of the phantom model, the consistency and execution of exposure techniques, the methods used for image quality assessment, and the overall process of image acquisition and analysis. Each domain was independently rated as having a low risk, some concerns, or a high risk of bias. An overall risk of bias judgment for each study was then made based on these domain-level evaluations. Any disagreements between the reviewers were resolved through discussion and mutual consensus.

## Result

### Study selection

**Fig. 1:**
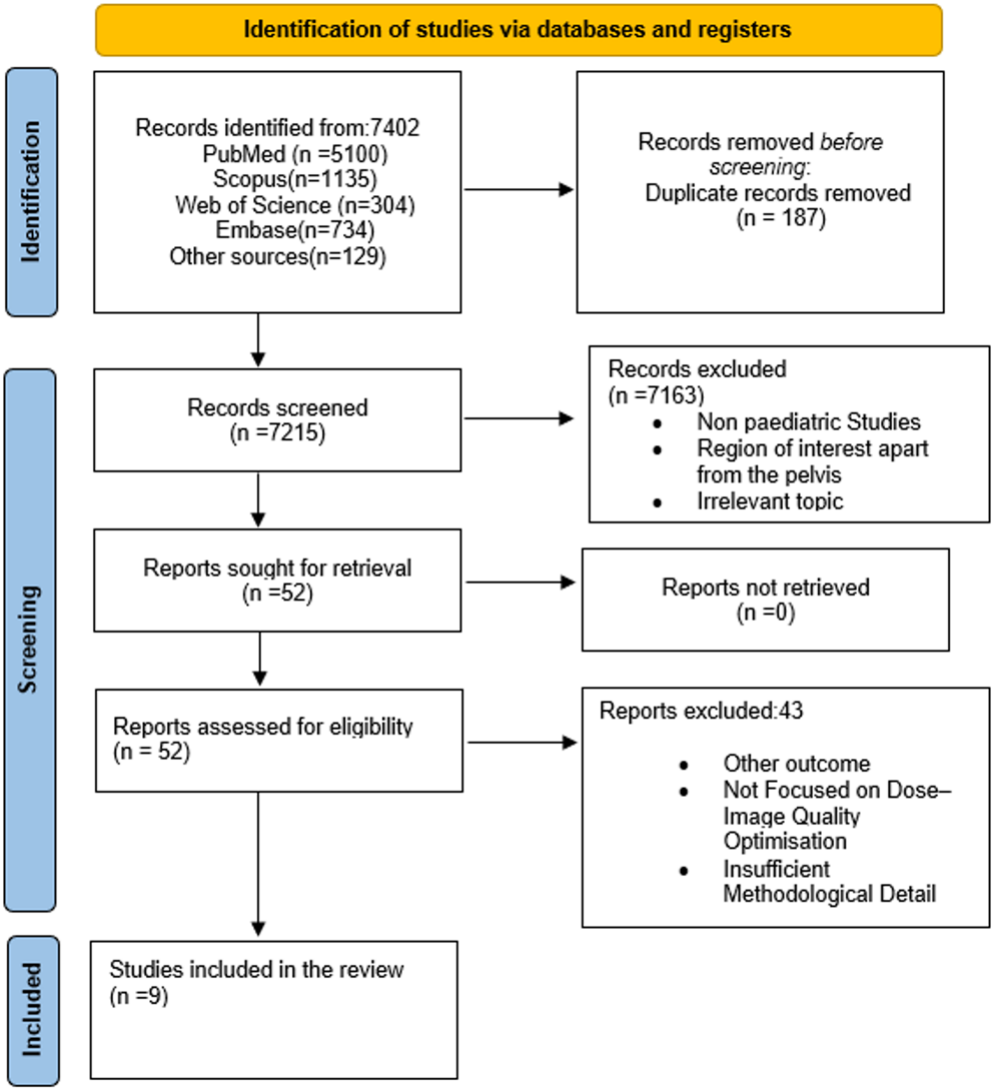
PRISMA flow diagram illustrating the study selection process for inclusion in the systematic review

This review systematically evaluated optimisation techniques in paediatric pelvic radiography, with particular focus on tube voltage (kVp), tube current-time product (mAs), source-to-image distance (SID), filtration, and grid application. A systematic literature search was conducted across four electronic databases, yielding a total of 7,402 records. After the removal of 187 duplicates, 7,215 articles remained for screening. During title and abstract screening, 7,163 articles were excluded because they did not meet the inclusion criteria, leaving 52 articles for full-text assessment. Of the 52 full-text articles retrieved from database searching, 43 were excluded due to reasons such as population mismatch, non-comparable imaging protocols, the absence of predefined outcome measures, or the inability to extract usable data for analysis. A total of 9 studies are included in this review(Fig. 1).

### Study characteristics

A total of nine studies published between 2004 and 2025 were included in this systematic review. Of these, seven were experimental studies [24–30] one was a conference proceeding [31] and one involved a human population [32]. Most investigations employed anthropomorphic phantoms to simulate pelvic anatomy and evaluate optimisation strategies in pelvic radiography. The studies were conducted across the UK [27, 28, 31], Ireland [24, 25], Lithuania [29], Switzerland [26], Iran [32], and Japan [30], reflecting a broad geographical distribution. Among the experimental studies, seven utilised a single paediatric phantom, whereas one study employed three different phantom models [25], contributing to heterogeneity in simulated patient anatomy due to variations in manufacturers and anatomical design. Ethical approval was generally not reported, as most studies involved non-human surrogates; however, the studies by Karami et al. (2017) [32] and Suzuki et al. (2025) [30] explicitly reported obtaining ethical approval. With respect to image quality assessment, both objective and subjective approaches were reported across the included studies. Objective quantitative indicators, such as the contrast-to-noise ratio (CNR) and signal-to-noise ratio (SNR), were reported in four studies [26, 29–31].In contrast, five studies predominantly employed subjective image quality assessment methods, including observer-based scoring systems and visual grading analysis. Although objective measures were incorporated in several investigations, heterogeneity in reporting and the limited availability of directly comparable quantitative data hinder robust cross-study comparisons [24, 25, 27, 28, 32].

The pelvic region was the primary imaging area of interest across all nine included studies. With respect to imaging systems, the majority of investigations utilised a single radiographic unit; however, Jreije et al.(2024) [29] conducted a comparative evaluation across eight digital radiography systems, offering broader insight into system-specific variability.The principal outcome measures assessed were effective dose (ED) and image quality (IQ). Additional dosimetric indicators included entrance surface dose (ESD), dose–area product (DAP), and system-specific exposure parameters, all contributing to the evaluation of radiation safety alongside diagnostic performance. Experimental exploration of technical factors encompassed tube potential (kVp), tube current–time product (mAs), source-to-image distance (SID), the use of copper or additional filtration, and the application of anti-scatter grids. A summary of the methodological and experimental characteristics of the included studies is presented in Table 1.

**Table 1:** Characteristics of the included studies

Author, Year, Country	Study Design	Phantom type	Subject	Number of Phantoms used	Modality	Region of Interest	Projection Type	Number of X-ray Systems used	X-ray model	Detector type used
Grondin et al. 2004 Ireland [24]	ES	RANDO Alderson anthropomorphic phantom	NA	1 Phantom	CR	Pelvis	AP	1	Siemens Polyphos 30 mol generator	Lanex Regular/Fast
M.L. Butler& Brennan 2009, Ireland [25]	ES	ATOM Paediatric Newborn ATOM Paediatric 5-Year-Old ATOM Adult Female	NA	3 Phantom	CR	Pelvis	AP	1	TOSRAD Toshiba Medical Systems	Agfa ADCC digital im age receptor plate
P. Brosi et al. 2011 Switzerland [26]	ES	PMMA blocks + CDRAD contrast–detail phantom	NA	1 Phantom	CR&DR	pelvis	AP	3	Philips PCR CosimaX& Philips Digital Diagnost	**CR**: Fuji ST-VI (single-sided), Fuji ST-BD (dual-sided) **DR**: CsI/a-Si flat-panel detector (Trixell Pixium 4600)
Tugwell et al.2014, UK [27]	ES	Anthropomorphic pelvis phantom	NA	1 Phantom	DR	Pelvis	AP	1	Wolverson Acroma X-ray	Flat-panel DR (CsI)
Lança et al.2014, UK [28]	ES	Rando SK250 sectional lower torso anthropomorphic phantom	NA	1 Phantom	CR	Pelvis	AP	1	Wolverson Arcoma X-ray	Agfa CR 35–43
Bloomfield et al.2014, UK [31]	ES	ATOM Dosimetry Verification Phantom, Model 705	NA	1 phantom	CR	Pelvis	AP	1	Siemens Polydoros IT 30/55/65/80 X-ray unit	Agfa CR
Karami et al. 2017, Iran[32]	PS	NA	Pediatric	NA	CR	Pelvis	AP	1	Varian Radiography system (UAS)	Konica REGIUS 210
Jreije et al.2024, Lithuania [29]	ES	(CIRS, US) MODEL 801-P phantom	NA	1 Phantom	DR	Pelvis	AP	8	GE Definium 6000	Flat panel detector (CsI)
									GE Discovey XR656	Flat panel detector (CsI)
									Philips CombiDiagnost R90	Flat panel detector (CsI)
									Shimadzu Sonialvision Safire	Amorphous selenium
									Shimadzu RADSpeed PRO	Flat panel detector (CsI)
									Siemens Luminos dRF	Flat panel detector (CsI)
									Siemens Ysio Max	Flat panel detector (CsI)
									Siemens Axiom Aristos MX	Flat panel detector (CsI)
Suzuki et al.2025, Japan [30]	ES	Newborn whole-body anthropomorphic phantom (PBU-80)	NA	1 Phantom	DR	Pelvis	AP	1	Shimadzu RAD Speed Pro radiography system	Fujifilm CALNEO Smart

**ES-**Experimental study, **PS**-Prospective study, **CR / DR-** Computed Radiography / Digital Radiography systems, **AP**-Anteroposterior projection

### Impact of technical factors on pelvic radiography

#### Tube potential (kVp)

An increase in tube potential (kVp), particularly when combined with an increased source-to-image distance (SID), was consistently associated with a reduction in effective dose, with image quality either maintained or only modestly affected. Grondin et al. (2004) demonstrated that increasing tube potential from 66 to 73 kVp while extending the SID from 100 cm to 130 cm reduced the effective dose from 0.60 mSv to 0.27 mSv, without compromising diagnostic image quality [24].Similarly, under automatic exposure control (AEC) conditions, effective dose decreased with increasing kVp, ranging from 0.22 to 0.30 mSv at 60 kVp, primarily due to automatic modulation of the tube current–time product (mAs), and no statistically significant deterioration in perceptual image quality was observed. In contrast, under manual (non-AEC) conditions, application of the 10 kVp rule across the 60–120 kVp range resulted in a reduction in effective dose from 0.37 mSv to 0.13 mSv (Table 2). Although observer performance declined from 94% at 60 kVp to approximately 80% at 90–120 kVp, and mean global image quality scores decreased from 73.9% to 49.0% in manual mode and from 77.2% to 48.0% in AEC mode, images remained diagnostically acceptable across all evaluated exposure settings (Table 3) [28]. Further evidence of the dose–image quality trade-off was provided by Suzuki et al. (2025) in infant hip imaging using a flat-panel detector. The introduction of additional copper filtration (0.1 mm Cu) at low tube voltages (40–55 kVp) reduced the entrance surface dose (ESD) from 13.8 µGy to 11.0 µGy (Table 2), while preserving clinically acceptable image quality. Objective image-quality metrics demonstrated an increase in signal-difference-to-noise ratio (SDNR) from 3.6 ± 0.1 at 40 kVp to 4.9 ± 0.1 at 55 kVp, accompanied by improvements in inverse image-quality figure (IQFinv), indicating enhanced contrast detectability at higher tube potentials. Despite these objective variations, subjective image quality remained consistently acceptable, with radiographer scores ≥ 3.6 and orthopaedic surgeon preference scores showing no statistically significant differences among the selected low-dose protocols (Table 6) [30]. System-dependent optimisation was further highlighted by Jreije et al. (2024), who evaluated pelvic imaging across eight digital radiography systems using an anthropomorphic phantom. Incremental increases in tube potential of 5 kVp from standard protocols resulted in dose–area product (DAP) reductions of 12–20% per increment, with cumulative reductions of up to 60–70% at 85–90 kVp, depending on the system (Table 2). These dose reductions were accompanied by only modest decreases in objective image quality, with CNR declining by 2–10% per 5 kVp increase, while subjective observer assessment revealed no perceived loss of diagnostic image quality up to 85–90 kVp for most systems, despite inter-system variability (Table 5) [29].

**Table 2:** Technical Parameters and Dosimetric Outcomes of the Included Studies

Study	kVp (S)	mAs (S)	AEC Used (S)	SID (cm) (S)	Filtration (S)	kVp (O)	mAs (O)		AEC Used (O)	SID (cm) (O)	Filtration (O)	Grid	Dose Metric	Standard Dose		Optimized Dose				Measurement Method
Grondin et al.[24]	66	NR	NO	100	2.5 mm Al	63–96	NR		NO	110–130	NR	NR	ED(mSv)	0.60		0.27				TLDs
M.L. Butler& Brennan [25]	60 (0y),	2 (0y)	NO	110	2.77 mm Al	60 (0y)	NR		NO	110	2.77 mm AL + 0.5–2.5 mm AL	NO	ESD(µGy) ED(µSv)	68.8 (0y) 588.6 (5y) 852 (15y)		64.8–48.4 (0y) 576.9-464.5 (5y) 790.3-619.9 (15y)		13.7–11.4 (0y) 89.4–81.8 (5y) 88.5–80 (15y)		Barracuda Multi-Purpose Detector & NRPB conversion
	73 (5y)	12.5 (5y)	NO	115		73 (5y)			NO	115	2.77 mm AL + 0.1–0.3 Cu	YES		14.0 (0y) 86.5 (5y) 90.3 (15y)		41.6–29.6 (0y) 379.6-256.7(5y) 567.2-406.8 (15y)		10.1–8.2(0y) 69.5–56.5 (5y) 77.7–71.6 (15y)		
	77 (15y)	AEC (15y)	YES	115		77 (15y)			YES	115	2.77 mm AL + + 0.1–0.3 Cu + 0.5–2.5 mm AL	YES				43.7–29.4 (0y) 380.3-272.1(5y) 548.8–383 (15y)		10.8–7.6 (0y) 70.4–58(5y) 76.8–64.4 (15y)		
P. Brosi et al. [26]	60 (0–1 y)	0.80	NO	120	2.5 mm Al	60 (0–1 y)	1.25	2.00	NO	120	0.1 mm Cu+1mmAl	OFF	ESD(µGy) (7,10&14 cm)	19.8	23.9	14.7		17.8		Keithley 35,050 A dosimeter.
														32.9	38.2	23.4		28.1		
														62.9	77.4	43.1		54.0		
	66 (2–7 y)	0.80				66 (2–7 y)	1.60	2.00			0.2 mm Cu+1mmAl					13.5		16.3		
																21.5		25.5		
																38.2		46.8		
	73 (8–18 y)	1.25				73 (8–18 y)	2.00	2.50			0.3 mm Cu+1mmAl					19.9		23.3		
																35.1		43.1		
											0.1,0.2,0.3 mm Cu		ED(µSv) 0y,1y,5y, 10y&15y)	3.5		3.4	3.3		-	
														1.9		2.0	2.0		-	
														4.7		4.6	4.6		4.5	
														9.6		8.9	8.7		8.6	
														8.2		7.8	7.7		7.6	
Tugwell et al. [27]	75	NR	YES	110	2.5 mm Al	75	NR		YES	90–140	NR	ON	ESD(mGy)	0.902		0.746				TLDs
													ED(mSv)	0.073		0.071				
Lança et al. [28]	60	NR	YES	110	3 mm Al	60–120	NR		NO	110	NR	ON	ED(mSv)	0.22–0.30		0.37 − 0.13				Monte Carlo (PCXMC)
Bloomfied et al. [31]	50–70	2.2-5	No	115	1 mm Al	50–70	2.2-5		NO	115	0.1–0.2 mm Cu	OFF	ED	Lower		40% reduction				PCXMC software
Karami et al. [32]	NR	NR	NO	100	3 mm Al	NR	NR		NO	130	3 mm Al	NR	ESD (µGy) (0-13y)	623		432				TLDs
Jreije et al. [29]	75–85	12.08–28.4	NO	115–120	NR	90	NR		YES	115–120	NR	ON	DAP(Gy·cm²)	0.6–1.04		0.15–0.51				DAP meter
Suzuki et al.[30]	50	2	NO	124–130	2.8 mm Al	40–55	2–5		NO	124–130	2.8 mm Al + 0.2 Cu	NR	ESD(µGy)	13.8		11				Ionization chamber

**S-**Standard Technique, **O-**Optimization Technique, **NR-**Not Reported, **ED-**Effective Dose, **ESD-**Entrance Surface Dose, **DAP-**Dose Area Product, **TLDs-**Thermo luminescent Dosimeters, **PCXMC -** Monte Carlo simulation, **DAP meter** - integrated device reading

AEC Used- Indicates whether Automatic Exposure Control (AEC) was enabled (“YES”) or manual exposure was employed (“NO”).Grid: “ON” denotes use of an anti-scatter grid. Filtration: Additional beam filtration used during acquisition (e.g., 2.5 mm Al, 0.1–0.2 mm

**Table 3:** Summary of image-quality assessment methods and imaging parameters used in classical pelvis optimisation studies

Study	IQ assessment methods	kVp (S)	SID (cm) (S)	Casset type (S)	Screen type (S)	Filtration (S)	kVp (O)	SID (cm) (O)	Casset type (O)	Screen type (O)	Filtration (O)	Subjective scoring / observers
Grondin et al. [24]	Anatomical criteria scoring (0–3 per criterion); panel of physicist + ≥ 2 radiographers	66	100	Aluminium front	Lanex Regular	2.5 mm Al	70	100–130	Carbon-fibre front	Lanex Fast	NR	No significant difference in IQ
M.L. Butler& Brennan [25]	Visual image quality assessment using predefined anatomical criteria	60 (0y), 73 (5y), 77 (15y)	100 (0y), 115 (5y,15y)	Agfa ADCC CR imaging plate	Photostimulable phosphor (CR)	2.77 mm Al	60 (0y), 73 (5y), 77 (15y)	100 (0y), 115 (5y,15y)	Agfa ADCC CR imaging plate	Photostimulable phosphor (CR)	2.77 mm AL + 0.5–2.5 mm AL, 2.77 mm AL + 0.1–0.3 Cu, 2.77 mm AL + + 0.1–0.3 Cu + 0.5–2.5 mm AL	All images scored ≥ 24/27 using nine anatomical criteria; most scored 27/27. No statistically significant differences in image quality were observed between baseline and additional filtration across all age groups
Tugwell et al. [27]	2AFC + 5-point Likert scale; 14 anatomical criteria adapted from European Guideline	75	110	Agfa CR	Computed Radiography plate	2.5 mm Al	75	90–140	Agfa CR	Computed Radiography plate	NR	No significant reduction in IQ at any SID (*p* = 0.967); 7 observers; ICC = 0.77 indicating strong agreement
Lança et al. [28]	2AFC visual grading; psychometric scale; 5-point Likert; anatomical criteria for pelvis	60	110	Agfa CR	CR image receptor	3 mm Al	60–120	110	Agfa CR	CR image receptor	NR	Image quality decreased slightly at high kVp but NOT statistically significant (*p* > 0.05 in most AEC modes); Manual mode IQ decreases from 94%→80%. 5 observers
Karami et al. [32]	Visual Grading Analysis (VGA) using European image quality criteria	NR	100	Imaging plate cassette (CR)	Photostimulable phosphor (PSP)	3 mm Al	NR	130	Imaging plate cassette (CR)	Photostimulable phosphor (PSP)	3 mm Al	Optimum image quality maintained; no image degradation

**2AFC -** Two-alternative forced-choice test, **NR**-Not Reported

**Table 4:** Image quality assessment using contrast–detail analysis under standard and copper-filtered exposure conditions in pediatric digital radiography

Study	IQ assessment methods	kVp/Thickness (S)	Filtration (S)	Mean IQFinv (S)			kVp/Thickness (O)	Filtration (O)	Mean IQFinv (O)		
				CR-ST	CR-BD	DR			CR-ST	CR-BD	DR
P. Brosi et al. [26]	Objective, contrast–detail analysis	60 kV/7 cm	2.5 mm Al	2.26	2.03	2.39	60 kV/7 cm	0.1mmCu	2.01	1.86	2.57
		66 kV/10 cm		1.49	1.50	1.87	66 kV/10 cm		1.59	1.42	2.02
		73 kV/14 cm		1.06	1.07	1.37	73 kV/14 cm		1.01	1.21	1.31
							60 kV/7 cm	0.2mmCu	2.17	2.10	2.20
							66 kV/10 cm		1.45	1.48	1.80
							73 kV/14 cm		0.94	1.03	1.31
							66 kV/10 cm	0.3mmCu	1.23	1.26	1.80
							73 kV/14 cm		0.99	0.99	1.23

**Table 5:** Objective and subjective image-quality assessment

Study	System	IQ assessment methods	kVp (S)	mAs (S)	SID (cm) (S)	Casset type (S)	Filtration (s)	CNR/SNR (S)	kVp (O)	mAs (O)	SID (cm) (O)	Casset type (O)	Filtration (o)	CNR/ SNR (O)	Subjective scoring/observers
Bloomfield et al. [31]		SNR, CNR (Objective)	50–70	2.2-5	115	Agfa CR	1 mm AL	NR	50–70	2.2-5	115	Agfa CR	0.1–0.2 mm Cu	NR	18/27 images (66.7%) demonstrated SNR and CNR equal to or higher than the lowest acceptable image-quality level
		2-Alternative Forced Choice (2AFC) (Subjective)													10 observers (5 experienced radiographers, 5 students); images scored twice; high intra- and inter-rater reliability (ICC > 0.79)
Jreije et al. [29]	GE Definium 6000	CNR (objective); 5-point Likert scoring (EC pelvis criteria)	80	17.7	115–120	DR flat-panel	-	1.6	65–90	-	115–120	DR flat-panel	-	↓40–44%	Slight agreement K = 0.05; IQ stable
	GE Discovery XR656		80	17.4	115–120	DR flat-panel	-	3.2	65–90	-	115–120	DR flat-panel	-	↓40–44%	Slight agreement K = 0.08; IQ stable
	Philips CombiDiagnost R90		85	12.08	115–120	DR flat-panel	-	1.6	65–90	-	115–120	DR flat-panel	-	↓40–44%	No agreement K = 0; IQ variable
	Shimadzu Sonialvision Safire		75	20.5	115–120	Direct Conversion	-	1.2	65–90	-	115–120	Direct Conversion	-	↓40–44%	Slight K = 0.11; IQ stable
	Shimadzu RADSpeed PRO		80	28.4	115–120	DR flat-panel	-	1.4	65–90	-	115–120	DR flat-panel	-	↓40–44%	No agreement K = 0; IQ variable
	Siemens Luminos dRF		77	16.2	115–120	DR flat-panel	-	1.8	65–90	-	115–120	DR flat-panel	-	↓40–44%	Fair K = 0.22; only system with visible IQ decline at 90 kVp
	Siemens Ysio Max		77	15.1	115–120	DR flat-panel	-	1.6	65–90	-	115–120	DR flat-panel	-	↓50%	Fair K = 0.30; IQ stable
	Siemens Axiom Aristos MX		77	18.3	115–120	DR flat-panel	-	2.4	65–90	-	115–120	DR flat-panel	-	↓34%	Fair K = 0.27; IQ stable

**CNR** -Contrast-to-Noise Ratio, **SNR**-Signal-to-Noise Ratio, **EC**-European Commission, **a-Se** - amorphous selenium, **κ (kappa) -**indicates inter-observer agreement strength for subjective evaluation

**Table 6:** Objective (SDNR, IQFinv) and subjective (RT and surgeon scoring) image-quality indicators under multiple low-dose pelvic imaging conditions in Suzuki et al. (2025)

Study	IQ assessment methods	kVp (S)	mAs (S)	SID (S)	Cassette (S)	Screen (S)	Filter (S)	SDNR (S)	RT Score (S)	Surgeon Score (S)	kVp (O)	mAs (O)	SID (O)	Cassette (O)	Screen (O)	Filter (O)	SDNR (O)	RT Score (O)	Surgeon Preference Score (O)
Suzuki et al. [30]	RT scoring (1–5), Scheffe paired comparison (Ura), Surgeon preference scoring, SDNR, IQFinv	50	2	130	FPD system	CsI FPD	2.8 mm Al + Cu 0.1 mm	SDNR 4.5 ± 0.2, IQFinv 3.8 ± 0.2	3.6	NA	40	2	130 cm	FPD system	CsI FPD	Cu 0	3.6 ± 0.1	3.6	0.04
											40	5				Cu 0.1	3.8 ± 0.1	3.6	0.03
											45	3.2				Cu 0.1	4.3 ± 0.1	3.7	0.01
											50	2				Cu 0.1	4.5 ± 0.2	3.6	0.03
											55	2				Cu 0.1	4.9 ± 0.1	3.6	0.04

**SDNR** -Signal-Difference-to-Noise Ratio, **IQFinv**- Inverse image-quality figure, **RT score** = Radiographer technical acceptability rating

#### Source-to-image distance (SID)

As shown in Table 2, increasing the source-to-image distance (SID) consistently provided dual benefits, achieving meaningful reductions in radiation dose while maintaining diagnostic image quality. Specifically, extending the SID from 100 cm to 130 cm reduced the mean effective dose from 0.67 mSv to 0.27 mSv [24]. Similarly, increasing the SID from 110 cm to 140 cm resulted in a reduction in entrance surface dose (ESD) from 0.902 mGy to 0.746 mGy, with a corresponding decrease in effective dose from 0.073 mSv to 0.071 mSv [27]. Importantly, image quality was largely preserved; visual grading analysis across fourteen anatomical criteria revealed no statistically significant deterioration, and inter-observer reproducibility was high (ICC = 0.77), despite a slight reduction in signal-to-noise ratio (SNR) at the longest SID. Consistent results were reported by Karami et al. (2017), who demonstrated that increasing the film-to-focus distance (FFD) from 100 cm to 130 cm in paediatric pelvic radiography (0–13 years), using a total filtration of 3 mm Al, led to a substantial reduction in entrance surface dose from 623 µGy to 432 µGy. This dose reduction was achieved without degradation of image quality, as visual grading analysis confirmed optimal diagnostic quality at both distances, with no statistically significant differences between images acquired at 100 cm and 130 cm FFD (Table 3) [32].

#### Additional filtration

The use of additional filtration was consistently associated with progressive reductions in both entrance surface dose (ESD) and effective dose (ED) compared with the baseline total filtration of 2.77 mm Al. The introduction of additional aluminium filtration (0.5–2.5 mm Al) resulted in a reduction in ESD from 68.8 to 48.4 µGy (0 years), 588.6 to 464.5 µGy (5 years), and 852 to 619.9 µGy (15 years), with corresponding reductions in ED from 13.7 to 11.4 µSv, 89.4 to 81.8 µSv, and 88.5 to 80.0 µSv, respectively. More pronounced dose reductions were observed with the addition of copper filtration (0.1–0.3 mm Cu), where ESD decreased to 41.6–29.6 µGy (0 years), 379.6–256.7 µGy (5 years), and 567.2–406.8 µGy (15 years), while ED was correspondingly reduced to 10.1–8.2 µSv, 69.5–56.5 µSv, and 77.7–71.6 µSv. When combined aluminium and copper filtration was applied, further reductions were achieved, with ESD values of 43.7–29.4 µGy (0 years), 380.3–272.1 µGy (5 years), and 548.8–383.0 µGy (15 years), and ED decreasing to 10.8–7.6 µSv, 70.4–58.0 µSv, and 76.8–64.4 µSv, respectively (Table 2). Importantly, these dose reductions were achieved without compromising diagnostic image quality, as observer-based assessments confirmed maintained diagnostic acceptability across all filtration conditions (Table 3) [25].Further evaluation of copper filtration in combination with exposure optimisation demonstrated additional dose–image quality trade-offs. The lowest ESD (11 µGy) was observed at 40 kVp and 5 mAs with 0.1-mm Cu filtration, whereas the highest ESD (31 µGy) occurred at 65 kVp and 3.2 mAs with 0.2-mm Cu filtration. Overall, ESD values remained within a narrow low range (11–13.8 µGy) across most exposure combinations; however, higher tube voltages were associated with increased uterine dose, highlighting trade-offs in dose distribution. Image quality analysis indicated that clinical acceptability could be preserved at lower-dose exposures, with five exposure combinations rated ≥ 3.5 (“good”), all acquired below 55 kVp and without 0.2-mm Cu filtration. Orthopaedic surgeons consistently preferred low-kVp exposures, particularly 40 kVp at 2.0 mAs, despite slightly lower objective image-quality indices. Objective metrics peaked at 55 kVp and 2 mAs with 0.1-mm Cu filtration (SDNR: 4.9 ± 0.1; IQFinv: 4.2 ± 0.2), while surgeon preference indicated that lower-kVp images remained subjectively most acceptable (Table 6) [30]. Consistent findings were reported by Brosi et al. (2011) for pelvic radiography, where the introduction of copper filtration (0.1–0.3 mm Cu) combined with compensatory mAs adjustment resulted in a 31–44% reduction in ESD compared with aluminium filtration alone (Table 2). However, for anteroposterior pelvic projections, this reduction in surface dose was not accompanied by a comparable reduction in effective dose, which remained largely unchanged due to increased beam penetration associated with filtration and mAs compensation. Importantly, objective contrast–detail analysis (IQFinv) demonstrated no consistent or statistically significant deterioration in image quality across increasing copper thicknesses, confirming that additional filtration effectively reduces superficial dose without compromising diagnostic acceptability (Table 4) [26]. Similarly, Bloomfield et al. demonstrated that additional copper filtration combined with optimised low-mAs techniques further reduced radiation dose while preserving image quality in paediatric AP pelvic radiography. Using 0.1–0.2 mm Cu filtration, effective dose was reduced by up to 40% compared with standard acquisition parameters, with 23 of 27 images (85.2%) exhibiting an effective dose equal to or lower than the reference image (Table 2). Despite reductions in mAs, visual image quality remained acceptable, with 13 images meeting predefined diagnostic acceptability criteria based on European image-quality standards. Objective assessments confirmed that SNR and CNR values for most low-dose images were equal to or greater than those of the lowest acceptable reference image, indicating that dose reductions achieved through the combined use of additional filtration and mAs optimisation did not result in clinically relevant loss of image quality (Table 5) [31].

#### Risk of bias in studies

**Fig. 2:**
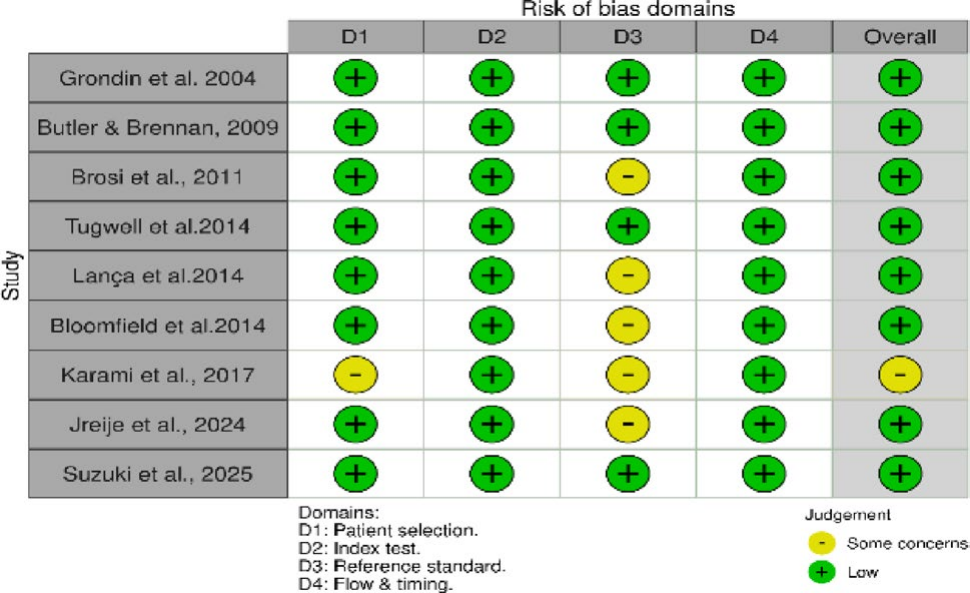
Quality assessment of the articles included in the review

The QUADAS-2 assessment indicated that most included studies exhibited an overall low risk of bias across the evaluated domains. Studies by Grondin et al. (2004), Butler and Brennan (2009), Tugwell et al. (2014), Suzuki et al. (2025), and Jreije et al. (2024) demonstrated low risk across participant selection (D1), index test (D2), and flow and timing (D4), reflecting the use of controlled phantom-based designs and predefined imaging protocols. Some concerns were primarily identified in the reference standard domain (D3) for studies by Brosi et al. (2011), Lança et al. (2014), Bloomfield et al. (2014), and Jreije et al. (2024), largely due to reliance on subjective image quality assessment methods without explicit reporting of observer blinding. Karami et al. (2017), the only study involving a human paediatric population, showed some concerns in participant selection (D1) in addition to the reference standard domain, reflecting limited reporting of recruitment methodology. A summary risk of bias assessment for the included studies is presented in Fig. 2.

## Discussion

This systematic review demonstrates consistent and converging evidence that optimisation of technical parameters in pelvic radiography can achieve substantial radiation dose reduction while maintaining diagnostically acceptable image quality. Across the included studies, increasing tube potential, extending source-to-image distance, and applying additional beam filtration were repeatedly associated with reductions in entrance surface dose and effective dose, with only modest or clinically acceptable effects on image quality. The most robust evidence was observed for additional filtration and SID extension, both of which consistently demonstrated reproducible dose-saving potential without compromising diagnostic performance. However, the predominance of phantom-based experimental studies necessitates cautious interpretation of these findings. While phantom models provide controlled conditions for evaluating optimisation strategies, they do not fully reflect real-world clinical practice. In paediatric imaging, factors such as patient motion, anatomical variability, positioning challenges, and cooperation levels can significantly influence both radiation dose and image quality. Therefore, although the direction of evidence is consistent, translation into routine clinical practice requires careful consideration and validation in real patient populations. From a clinical perspective, the findings of this review support the adoption of practical optimisation strategies in routine paediatric radiography. These include the use of higher kVp techniques combined with appropriate mAs reduction, extension of SID where feasible, and the incorporation of additional beam filtration to reduce superficial dose. As most evidence is derived from phantom-based studies, clinical implementation should be approached with caution and validated within local practice. Attention should be given to automatic exposure control (AEC), as default settings are often designed for adult imaging and may lead to unnecessary radiation exposure in paediatric patients. Paediatric-specific AEC calibration, appropriate chamber selection, and regular protocol audits are therefore essential to ensure dose optimisation and maintain diagnostic image quality [16–19] .

One of the most compelling findings across the reviewed literature was reported by Grondin et al., who demonstrated that a combined strategy of increasing tube potential and extending the focus-to-imaging plate distance resulted in an effective dose reduction of up to 63%, without a statistically significant decline in image acceptability [24]. This finding provides strong evidence that geometric and exposure-based optimisation strategies can be synergistically applied to achieve substantial dose savings. Similar trends were observed by Lai et al., who reported dose reductions of up to 59.5% without copper filtration and 43.7% with additional filtration, with the magnitude of reduction dependent on filter thickness and exposure configuration [33]. Although filtration markedly reduced patient dose, these authors also reported significant reductions in contrast-to-noise ratio (CNR), highlighting the need to balance dose efficiency against diagnostic image quality. Extending the source-to-image distance (SID) consistently emerged as a reliable and reproducible optimisation strategy. Tugwell et al. [27] demonstrated dose reductions of 17.3% for entrance surface dose (ESD) and 3.7% for effective dose under automatic exposure control (AEC), with substantially greater reductions exceeding 40% for both metrics when AEC was not employed. This suggests that AEC target exposure levels in the evaluated system may have been calibrated conservatively, resulting in suboptimal dose efficiency. The potential for further optimisation through refinement of AEC parameters, rather than simple activation or deactivation, was also emphasised. However, detailed assessment of AEC behaviour remains limited due to insufficient reporting of key parameters such as target detector dose, chamber selection, and backup mAs thresholds. The variability in AEC performance across systems was further underscored by Jreije et al., who demonstrated marked inter-system dose variability across eight digital radiography platforms, attributable to differences in detector sensitivity, beam filtration, generator performance, and manufacturer-specific AEC algorithms [29]. These findings support the need for paediatric-specific AEC calibration, rather than reliance on adult default settings. Phantom-based evidence suggests that lowering AEC target detector dose could yield dose reductions of 25–40%, albeit with increased quantum noise, reinforcing the need for careful optimisation tailored to patient size and clinical indication. The effectiveness of SID extension was further validated in clinical and experimental contexts. Karami et al. [32] demonstrated that increasing the film-to-focus distance from 100 cm to 130 cm in paediatric pelvic radiography resulted in statistically significant reductions in ESD without compromising diagnostic image quality, findings that were corroborated by visual grading analysis. Similarly, Ward et al. confirmed that longer SID reduces dose in accordance with the inverse square law, with no significant deterioration in image quality [34]. Importantly, this clinical evidence supports the feasibility of implementing optimisation strategies in routine practice. In addition to the findings of Karami et al. [32], paediatric imaging initiatives such as the “Image Gently campaign” emphasise the practical application of dose optimisation techniques, including adjustment of exposure parameters based on patient size, minimisation of mAs, and avoidance of unnecessary radiation exposure through protocol adaptation [18, 19]. These recommendations align closely with the optimisation strategies identified in this review, reinforcing their clinical applicability.

The clinical effectiveness of the 10 kVp rule was consistently demonstrated in studies by Lança et al. and Allen et al., where incremental increases in tube potential combined with proportional reductions in mAs achieved dose reductions of up to 64.9% in manual mode and 36% across large phantom datasets, without significant loss of perceived diagnostic quality [28, 35]. Although subjective image quality scores declined at higher kVp values, diagnostic acceptability remained intact, supporting the principle that modest reductions in contrast can be tolerated in exchange for substantial dose savings. Equipment variability emerged as a critical factor in optimisation. Sun et al. demonstrated that direct digital radiography systems provide superior image clarity and lower noise compared with computed radiography at equivalent dose levels [36], while Jreije et al. reported substantial inter-system variability in contrast-to-noise ratio (CNR) despite similar exposure parameters [29]. These findings highlight the importance of system-specific optimisation protocols and ongoing quality assurance to prevent “dose creep,” a recognised risk associated with the wide dynamic range of digital detectors. A recurring observation across studies was the stronger dependence of image noise on mAs rather than kVp, while dose reduction was more effectively achieved through kVp modulation. This supports the clinical strategy of employing higher kVp techniques with corresponding mAs reduction, consistent with the ALARA principle and the concept of producing images that are “as good as needed” rather than “as good as possible.” Notably, divergence between objective image quality metrics (CNR, SNR, IQFinv) and subjective observer assessments was evident across multiple studies. While quantitative measures often showed declines under dose-optimised conditions, clinicians frequently rated the images as diagnostically acceptable. Jreije et al. [29] reported low inter-observer agreement, whereas Suzuki et al. demonstrated consistent clinical preference for low-dose protocols despite lower objective scores [30]. These findings emphasise the importance of integrating both quantitative and clinically driven evaluation methods. The role of additional filtration in dose reduction was strongly supported. Butler and Brennan demonstrated ESD reductions of up to 62.9% and effective dose reductions of up to 46.4% without significant loss of image quality [25], while Brosi et al. confirmed that copper filtration effectively reduces surface dose, although reductions in effective dose may be less pronounced due to increased beam penetration [26]. Similarly, Suzuki et al. reported dose levels well below current diagnostic reference levels, suggesting that existing DRLs may not fully reflect optimisation potential achievable with modern imaging systems [30]. However, heterogeneity in imaging systems, exposure protocols, dosimetric endpoints, and image quality assessment methods limited direct quantitative comparison and precluded meta-analysis. This highlights the need for standardised imaging protocols and reporting frameworks.

The optimisation strategies behave differently in paediatric pelvic radiography compared with adult imaging due to the difference in body size and thickness of the anatomical region. Children are more radiosensitive and may receive higher organ doses for equivalent exposure settings due to reduced body attenuation and increased tissue sensitivity [37]. Furthermore, optimisation strategies such as tube potential selection and exposure parameter adjustment are highly dependent on patient size and therefore require paediatric-specific adaptation rather than direct application of adult protocols [18]. In addition to patient-related factors, anatomical region plays a critical role in optimisation. The pelvic region contains several radiosensitive organs, including gonads and bone marrow, which contribute significantly to radiation risk. Consequently, optimisation strategies that may be acceptable in lower-dose examinations, such as extremity imaging, may not be directly transferable to pelvic radiography. A recent multicentre study evaluating national diagnostic reference levels (NDRLs) for paediatric pelvic radiography reported a mean entrance surface dose (ESD) of 0.69 mGy. Age-specific DRLs were identified as 0.265 mGy (0 to < 1 year), 0.382 mGy (1 to < 5 years), 0.704 mGy (5 to < 10 years), and 0.995 mGy (10 to ≤ 15 years), indicating that radiation dose increases with age and is influenced by patient size and body thickness [38].

Overall, the findings of this review demonstrate the effectiveness of optimisation strategies using phantoms in reducing radiation dose without compromising diagnostic image quality, their clinical implementation requires careful adaptation to real-world paediatric imaging conditions. Practical application should involve system-specific protocol optimisation, paediatric-focused AEC calibration, and regular quality assurance processes to ensure consistent and safe use. From a clinical perspective, the impact of dose optimisation is dependent on the clinical condition being examined. In paediatric pelvic radiography, where assessment of fractures, developmental dysplasia of the hip, and alignment is primarily based on bony structures, modest reductions in contrast or increased noise may be acceptable, provided key anatomical landmarks remain clearly visible. However, excessive dose reduction may compromise subtle diagnostic details, highlighting the need to balance optimisation specific to the clinical condition. Furthermore, the predominance of phantom-based evidence and variability across imaging systems highlights the need for well-designed multicentre patient-based studies with standardised methodologies, which will reduce inter-centre variability, making quantitative comparison convenient by minimising the heterogeneity [38]. Further, such studies are essential to validate current findings, improve generalisability, and support the development of robust clinical guidelines for paediatric pelvic radiography.

### Study limitations

This review has several limitations that should be considered when interpreting the findings. Most included studies remained phantom-based, which limits the direct translation of results to routine clinical practice where patient anatomy, positioning variability, and motion particularly in paediatric populations can substantially influence both radiation dose and image quality. While phantom studies are valuable for controlled optimisation research without ethical constraints, they cannot fully replicate real-world clinical conditions. Only one included study involved a human population, restricting the strength of clinical inference. Most investigations were conducted using single imaging systems, thereby limiting the generalisability of findings across different detector technologies, manufacturers, and system-specific configurations. In addition, anthropomorphic phantoms were not consistently linked to clearly defined paediatric age or size categories, preventing meaningful subgroup analysis across clinically relevant age groups such as neonates, infants, and older children. Consequently, age-dependent optimisation effects could not be adequately assessed. Image quality assessment across studies was predominantly subjective, relying on visual grading or observer scoring by a limited number of reviewers. Although some studies reported acceptable inter-observer agreement and incorporated objective metrics such as CNR, SNR, or IQFinv, these measures were inconsistently reported, introducing potential observer bias and limiting cross-study comparability. The included studies demonstrated variability in research objectives and optimisation tasks, which contributed to heterogeneity and limited direct comparability across findings. Furthermore, heterogeneity in exposure protocols, dosimetric endpoints, and reporting of automatic exposure control (AEC) parameters restricted direct quantitative comparison and precluded meta-analysis.

### Future research priorities

Future research should prioritise well-designed, multicentre clinical studies to validate phantom-based optimisation strategies in real-world paediatric settings. Studies should employ standardised, age- and size-stratified phantoms or patient cohorts, harmonised dosimetric endpoints, and combined objective and subjective image quality assessments. Greater emphasis is also needed on systematically evaluating AEC behaviour, including the impact of target detector dose, chamber selection, and backup mAs thresholds across different manufacturers. Such research would strengthen the evidence base, improve generalisability, and support the development of robust, clinically applicable optimisation protocols for paediatric pelvic radiography.

### Clinical implications

The findings of this review support the routine implementation of dose-optimisation strategies in paediatric pelvic radiography, including source-to-image distance (SID) extension, kVp optimisation with appropriate mAs reduction, judicious use of automatic exposure control (AEC), and additional beam filtration. When appropriately applied, these strategies can achieve meaningful reductions in radiation dose without compromising diagnostic image quality. The results highlight the importance of system-specific protocol optimisation, as reliance on default manufacturer settings particularly for AEC may lead to unnecessary radiation exposure. Radiographers and medical physicists should therefore collaborate to tailor exposure parameters to paediatric anatomy, ensuring image quality is “as good as needed” rather than maximised.

## Conclusion

This systematic review indicates that dose optimisation strategies such as increased kVp, extended source-to-image distance, and system-specific parameter adjustments can substantially reduce radiation dose in pelvic imaging without compromising image quality. However, the majority of included studies were phantom-based; the findings should be interpreted cautiously, acknowledging the limited clinical applicability. Further patient-based studies are warranted to validate these optimisation strategies under real-world conditions.

## Data Availability

All data generated or analysed during this study are included in this published article.
